# Multiple Effects of the Second Fluid on Suspension Viscosity

**DOI:** 10.1038/srep16058

**Published:** 2015-11-02

**Authors:** Jie Zhang, Hui Zhao, Weifeng Li, Menghan Xu, Haifeng Liu

**Affiliations:** 1 Key Laboratory of Coal Gasification and Energy Chemical Engineering of Ministry of Education, East China University of Science and Technology, P.O. Box 272, No. 130 Meilong Road, Shanghai 200237, People’s Republic of China; 2 Shanghai Engineering Research Center of Coal Gasification, East China University of Science and Technology, Shanghai 200237, People’s Republic of China

## Abstract

Previous research has shown that adding a small amount of a second immiscible fluid to particulate suspension can result in a significant influence on viscosity. In this study, the effects of the second fluid addition over a small dosage range on the rheological properties of particle suspension were investigated. As the dosage of the second fluid was increased, the viscosity and yield stress initially decreased then increased and finally decreased again. The microstructure of the suspension was observed using a confocal laser scanning microscope (CLSM) and showed three different states with the increasing dosage of the second fluid: a dispersive sate, cluster state and cell state in sequence. The presence of these states interpreted the non-monotonic trend of viscosity and yield stress in the suspensions.

The rheology of suspension is a complex function of the interactions between the particles, including van der Waals forces, electrostatic forces, steric interactions, hydrodynamic interactions, and Brownian forces^1^,^2^,^3^,^4^,^5^. In recent years, the capillary force, which also plays an important role in suspension rheology, has attracted increasing attention^6^,^7^,^8^,^9^. The addition of a small amount of a second immiscible fluid can dramatically change the macroscopic properties of the suspension. Koos and Willenbacher found that the second fluid works independent of whether the second fluid wets the particles better or worse than the primary fluid^10^. It has been shown to transform a viscous or weakly elastic fluid into a highly elastic or gel-like material^11^,^12^. Mixtures in which the second fluid preferentially wets the particle are referred to as pendular state suspensions, while capillary state suspensions are characterized by a preferentially nonwetting fluid as the second fluid in the mixture^10^,^13^,^14^. In the both states, the second fluid creates a network of interconnected particles.

Different from results reported by Koos *et al.*^10^, using a dispersant and pre-coating preparation, Xu *et al.* found that a second fluid could greatly decrease the viscosity and yield stress of suspensions^15^. The second fluid works as an adhesive by coating the particle surface and forming a thin hydrophobic film. The resulting particles have thinner hydration layers and are better dispersed in primary fluid with the dispersant, thus preventing the formation of liquid bridges. Previous research has examined the behavior of the viscosity and yield stress over a small dosage range of a secondary fluid. However, the effects of a second fluid applied over a wider dosage range on the viscosity and yield stress are not well understood. In the present study, particles of hollow glass bead (HGB, average diameter of 42 μm), polyethylene (PE, average diameter of 51 μm), and bituminous coal (BC, average diameter of 20 μm) were applied in experiments, with dispersant and different addition methods and different amounts of the second fluid. The main objective was to observe the microstructure in suspensions and elucidate the mechanism. The trend obtained unifies the different discoveries from Koos *et al.*^10^ and Xu *et al.*^15^, appearing to be a universal feature for particle suspensions.

## Results

### Influence of the second fluid on suspension viscosity and yield stress

**Figure 1:**
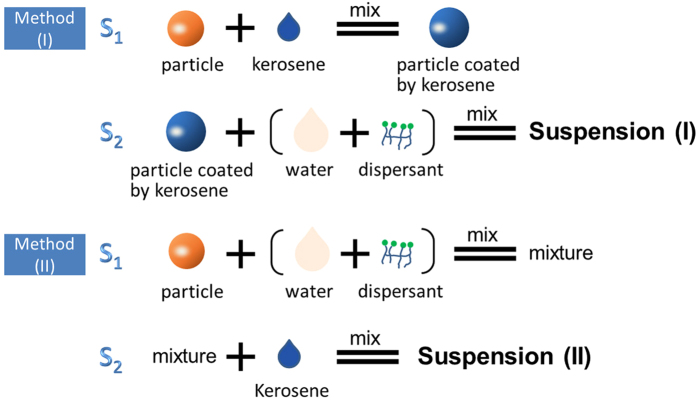
Schematic diagram of two preparation methods used in this study.

In the all particle suspensions, water is denoted as the primary fluid, while a small amount of kerosene is denoted as the second fluid, immiscible with the primary fluid. The all suspensions were composed of particles, water, a little amount of dispersant and kerosene (or without kerosene). Dispersant possess both hydrophobic and hydrophilic groups, i.e. it is a surfactant. Methyl naphthalene sulfonate formaldehyde condensation (MF) was chosen as the dispersants in this paper, which has a polymer chains and negative charge. Two preparation methods were used in this study (the schematic is shown in Fig. 1): method (I), in which particles were pre-coated with kerosene to form composite particles, and then mixed with water and dispersant evenly; and method (II), in which particles, water and dispersant were mixed evenly, and then kerosene was added to the water suspension followed several minutes agitation. The solubility of MF (dispersant) in kerosene is less than 10^−5^. The solubility of kerosene in the aqueous solution of MF (0.754 wt%) is less than10^−4^, and that of kerosene in water is less than 10^−5^. So the solubilization effect of MF for kerosene in water is insignificant.

**Figure 2:**
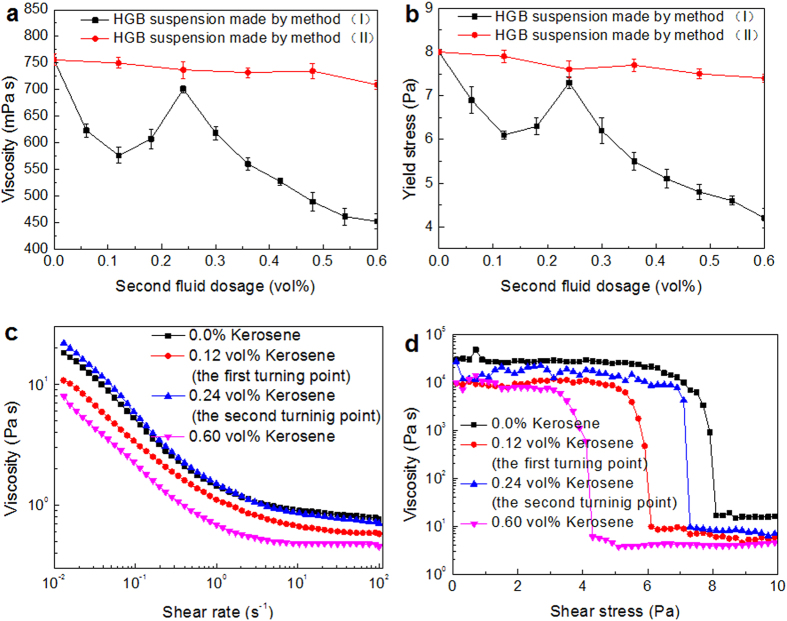
Effect of the addition of second fluid on the rheology of HGB suspension. Viscosity (**a**) at a shear rate of 100 s^−1^, and yield stress (**b**) trends with various second fluid dosages according to different preparation methods; Flow cures [(**c**,**d**)] by method (I) at the first and second turning points, and a larger dosage of second fluid. Error bars in (**a**,**b**) indicate repeatability error.

HGB in water were used as a model system to evaluate the performance of the second fluid in suspension. The viscosity and yield stress trend are shown in Fig. 2a,b, respectively. The solid loading in all suspensions was 61.0 vol% (43.0 wt%) with 1.0 wt% of dispersant (dry solid basis). For method (I), an increase in the second fluid content from 0 to 0.12 vol% (dry particle basis) resulted in a decrease in the viscosity from 756 mPa s to 576 mPa s. The viscosity then began to increase gradually to 701 mPa s until the second fluid reached 0.24 vol%. At the second fluid content exceeding 0.24 vol%, viscosity began to decrease rapidly. The viscosity decreases to 452 mPa s at the second fluid dosage of 0.60 vol%. The yield stress of suspensions was measured according to the procedure described by Moller P.C.F *et al.*^16^,^17^. For stresses smaller than that of the critical stress, the viscosity becomes so high that buildup of structure is prevented from destruction. On the other hand, for a stress slightly higher than that of the critical stress, destruction of the microstructure occurs, causing viscosity to gradually decrease and reach a low steady-state value. The yield stress exhibited similar variation trend over the same dosage region. For illustration, we arbitrarily termed the both break points in the secondary fluid content, 0.12 vol% and 0.24 vol% in HGB suspension, as “the first turning point” and “the second turning point”, respectively. The rheological curves and the measured yield stress for the suspension at different points are shown in Fig. 2c,d. PE and BC particles in water were also examined. Using method (I), the two particle suspensions displayed similar trends in viscosity and yield stress (see Supplementary Fig. S3 and S4).

Suspensions prepared via method (II) displayed a different trend compared to those prepared by method (I). For HGB suspensions, the apparent viscosity did not vary significantly as the dosage of the secondary fluid was increased from 756 mPa s (the second fluid 0 vol%) to 709 mPa s (the second fluid 0.60 vol%) (Fig. 2a). Similarly, no discernible change in viscosity was observed for PE and BC suspensions (Supplementary Fig. S3 and S4).

### Effects of the second fluid on contact angle

**Figure 3:**
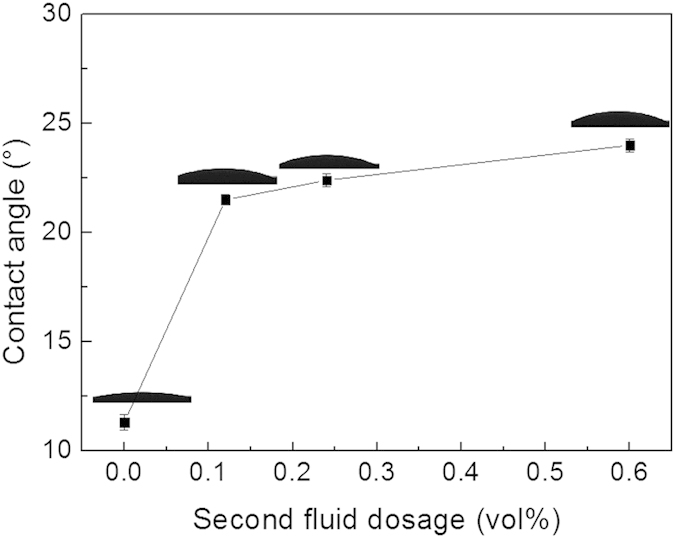
Effect of the second fluid on the contact angle of HGB particles. HGB without modification, θ = 11.3°; at the first turning point, θ = 21.5°; at the second turning point, θ = 22.4°; at one larger dosage of the second fluid, θ = 24.0°. Error bars indicate repeatability error.

In order to evaluate changes in the hydrophobicity of the particle surface modified by the second fluid through method (I), the contact angles of the composite particles at both turning points in air were measured. As shown in Fig. 3, the contact angle of HGB increased from 11.3° (blank sample) to 21.5° at the first turning point, and then increased slightly. The PE and BC particles displayed similar behavior upon addition of the second fluid (Supplementary Fig. S5 and S6). These results suggest that the hydrophobicity of particles improves considerably before the first turning point, which accounts for the observed decrease in viscosity and yield stress. After that, the hydrophobicity of particles increased very slowly, and did not display non-monotonic trend over the entire dosage range.

### Microstructures of suspensions containing the second fluid

**Figure 4:**
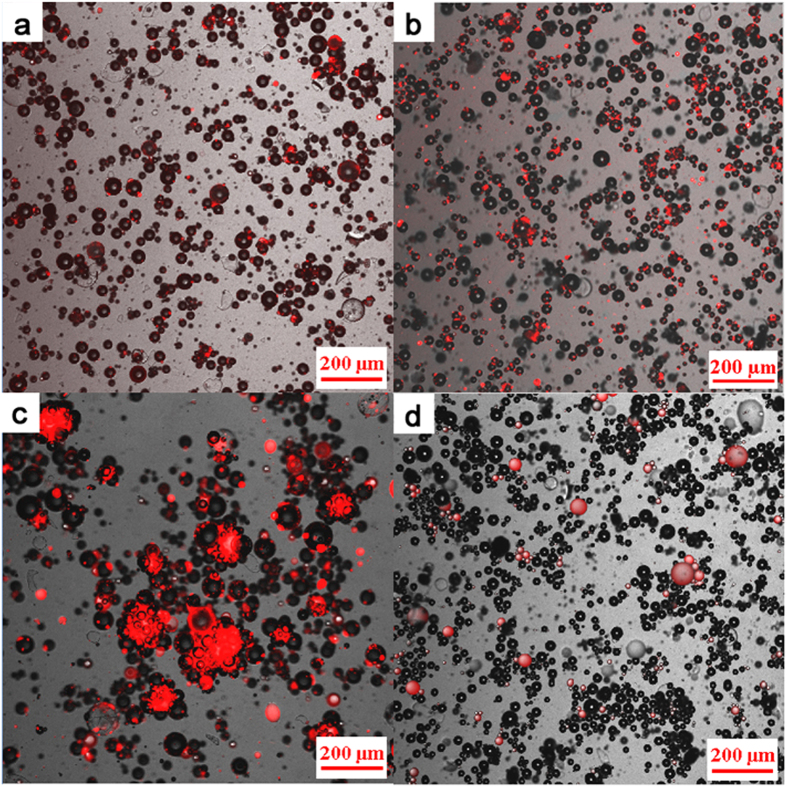
Microscopic images of HGB with the second fluid in different adding methods: (a–c), the first turning point, the second turning point and a larger dosage of second fluid made by method (I), respectively; (**d**) suspension made by method (II).

The microstructures of suspensions prepared through different methods were observed using a CLSM (Fig. 4). Hydrophobic fluorescent dye [DiIC1(5) iodide, Fanbo Biochemicals Co. Led.] was used to highlight the location of kerosene by regions red in color. The suspension microstructure at the first turning point, the second turning point, and at a larger dosage (0.60 vol%) prepared by method (I), are shown in Fig. 4a–c, respectively. The red regions in Fig. 4a indicate that particles were well dispersed after coating with a small amount of secondary fluid. As shown in Fig. 4b, the second fluid allowed the composite particles to form connected lines and a network structures. Figure 4c displays that cells are formed.

**Figure 5:**
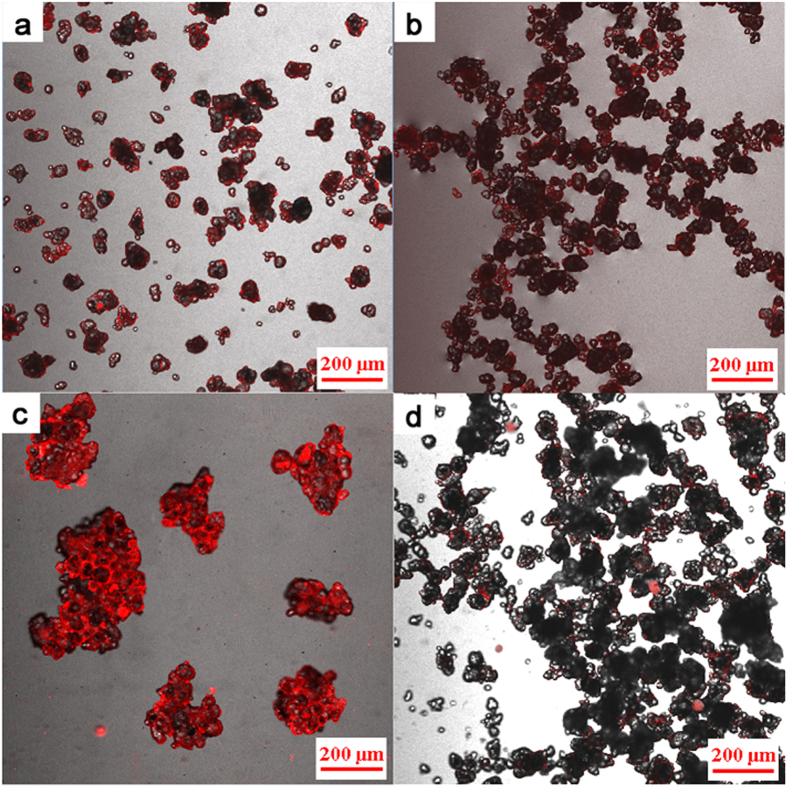
Microscopic images of PE with the second fluid in different adding methods. (**a**–**c**), the first turning point, the second turning point and a larger dosage of second fluid made by the method (I), respectively; (**d**) suspension made by method (II).

The behavior of the secondary fluid in HGB suspension prepared by method (II) is shown in Fig. 4d. The red dots of various sizes around the particles indicate that the second fluid existed in a free state. At different critical points and through different preparation method, the microstructures of PE and BC suspensions have similar behaviors like those of HGB suspension. They confirm that the addition method and amount of second fluid greatly affect the microstructure of particle suspension (Fig. 5, Supplementary Fig. S7).

## Discussion

**Figure 6:**
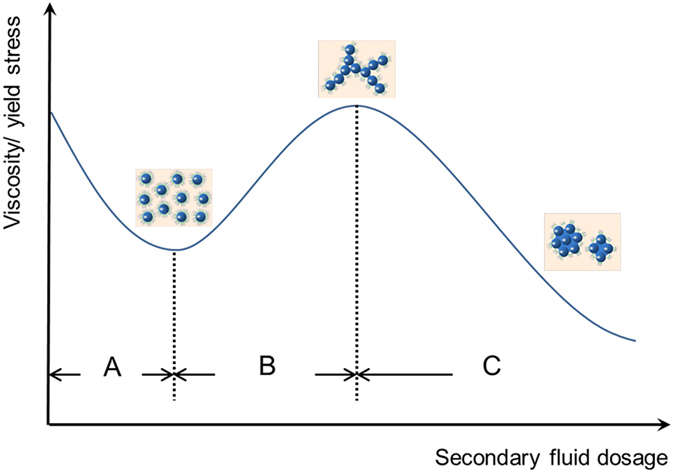
Viscosity and yield stress trends. With the increasing dosage of the second fluid (method (I)), the viscosity and yield stress initially decreased and then increased, and lastly decreased again.

**Figure 7:**
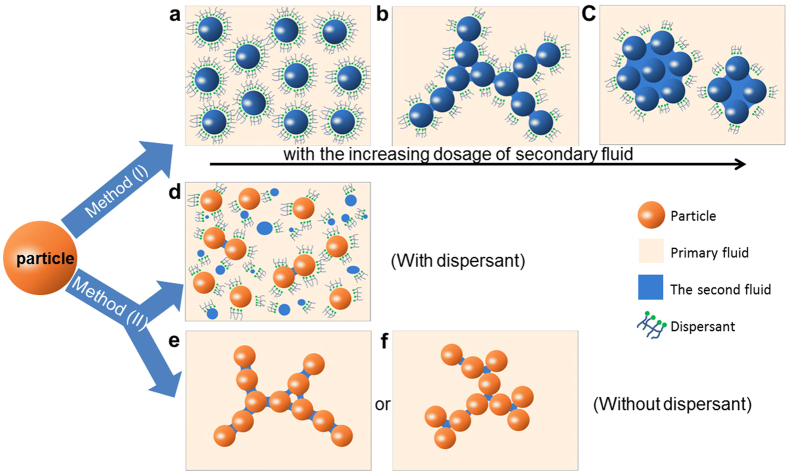
Schematic diagram of the influence of second fluid on particle suspension. (**a**–**c**), diagrams of particle morphology prepared by method (I) in “dispersive state”, “cluster state”, and “cell state”, respectively. (**d**), diagram of particles morphology prepared by method (II); (**e**,**f**) pendular state and capillary state interpreted by Koos *et al.*^10^, respectively.

As the dosage of the second fluid was increased in method (I), the viscosity and yield stress of different particle suspensions displayed similar trends, namely an initial decrease followed by an increase and then a rapid decrease (Fig. 6). The solid loading of suspensions remained the same, counting out the volume of the second fluid. Compared with the blank sample (without the second fluid), no obvious change was observed when the second immiscible fluid was replaced by the same volume of water by method (I). It suggested that the second fluid has a significant effect on the microstructure of suspension. This hypothesis was confirmed by characterization of the existence form of the secondary fluid (Fig. 4) and by measuring the hydrophobicity of composite particles (Fig. 3). Before the first turning point, the secondary fluid exhibited a decrease in viscosity (stage A in Fig. 6) due to the formation of hydrophobic films on the particle surface and the development of surface hydrophobicity. This composite structure disrupted the interactions between the particles and water, resulting in a thinner hydration layer^15^. The hydrophobic film also favored the adsorption of hydrophobic groups of dispersant through hydrophobic interaction. This resulted in spreading of polymer chains of dispersant and the development of steric and electrostatic repulsion among particles^18^,^19^,^20^ Thereby, the dispersion and stability of particles improved and pendular bridges were avoided^21^. This state was referred to as the “dispersive state” (Fig. 7a).

The kerosene content varied between the first and the second turning points, which induced an increase in suspension viscosity (stage B in Fig. 6). This can be attributed to the increasing dosage of the secondary fluid, which results in composite particles readily adhering together through hydrophobic interaction to against steric and electrostatic repulsion from the dispersant. Liquid bridges were formed and the number of capillary interaction sites increased gradually, which allowed particles to connect first into lines and finally into a stable network structure (Fig. 7b). The network can trap water inside, thus reducing the flowability of the suspension. This state was termed “cluster state”, and characterized by increasing viscosity and yield stress, which reached maximum values at the second turning point.

Upon further increase in the dosage of the second fluid, the viscosity and yield stress were found to decrease again. This decrease may be attributed to an increase in the volume of liquid bridges with limited change on the number of liquid bridge. Network structures were found to become increasingly loose and no longer display a significant yield stress. The structures were easily broken into cells through agitation (Figs 4c and 7c). Compared with the particles, these cells were larger in size, with wider particle size distributions, which contributed to the decrease in viscosity^22^,^23^. The cells, which can be considered deformable elastic particles, helped to further reduce viscosity^24^,^25^,^26^. Compared to the network structure, cells have a smaller area in contact with water and therefore release more free water, resulting in a dramatic decrease in viscosity (stage C of Fig. 6). This state is termed “cell state”. No sedimentation or phase separation was observed, in contrast to reports by Heidlebaugh *et al.*^27^. This difference is likely attributable to the reduction in interfacial tension by the dispersant.

In suspensions prepared by method (II), the second fluid failed to form hydrophobic films and existed in the form of micro-droplets. This state is termed “isolated state” (Fig. 7d). The existence of this state is difference than the pendular and capillary states described by Koos *et al.*^10^ (Fig. 7e,f). Their study also used method (II), but without dispersant. The dispersant decreases the interfacial tension between the two fluids, preventing the formation of liquid bridge and network structures. Many dispersants also tend to be absorbed onto the micro-droplets, resulting in steric hindrance which favors particle dispersion. Overall, the second fluid was found to have minimal influence on suspensions prepared by method (II).

## Conclusions

In this study, multiple effects of the second fluid addition on the rheology of particle suspensions containing dispersant were investigated. In method (I), the second fluid pre-coated particles and dramatically changed the rheological properties. When the dosage of the second fluid was increased, the viscosity of the suspension initially decreased and then increased, and lastly decreased again. The yield stress followed a similar trend. Three states (dispersive state, cluster state, and cell state in sequence) were observed by the characterizations, and found to follow the non-monotonic trends. The second fluid was found to reduce viscosity through the formation of hydrophobic films. A further increase in dosage resulted in an increase in viscosity, as composite particles adhered together to form liquid bridges and network structures. Higher dosages of the second fluid were found to rapidly reduce the viscosity due to the formation of cells, which exhibited lower hardness and wider size distributions. The trend obtained unifies the different discoveries from Koos *et al.*^10^ and Xu *et al.*^15^, appearing to be a universal feature for particle suspensions.

Findings from this study suggest that method (I) may be a promising technology for industrial applications, due to its convenience and propensity to reduce the viscosity of suspensions. The viscosity can be selectively tuned over a narrow range of second fluid dosages. One such application for method (I) may be in the preparation of highly concentrated coal water slurries used in clean coal technology. This pre-coating method of the second fluid exploits a new way for industrial production.

## Methods

### Materials

Hydrophilic hollow glass beads (HGB) were purchased from Suzhou Zeer Chemical Products Company (Jiangsu). Polyethylene (PE) was obtained from Yilufa Plastic Material Sales Department (Zhangmutou, Dongguan). Bituminous coal (BC) originated from Inner Mongolia. Methyl naphthalene sulfonate formaldehyde condensation (MF) was chosen as the dispersant, and the molecular structure is shown in Supplementary Fig. S2. Industrial kerosene was used as the second fluid.

HGB and PE particles were dried in an oven at 70 °C for 24 h. BC samples were dried at 105 °C for 24 h, and then comminuted using a ball mill. The resulting BC particles were then sieved through 40 and 200 mesh sieves to obtain two particle size distributions. Coarse and fine BC particles were mixed in a mass ratio of 6:4 for use in experiments.

### Sample preparation

The blend of 1.0 wt% dispersant (dry solid particle basis) and weighted deionized water was added into HGB, PE, and BC, respectively. Suspensions were mixed for 20 min by vortex at 1000 rpm.

As the second fluid, kerosene was added to particle mixtures using two methods: (I) First, kerosene was added into the dry solid particles in a flask and stirred for 30 min to ensure homogeneity. Particles coated by the second fluid were called composite particles. Then the composite particles were mixed with dispersant and deionized water for the final suspensions; (II) First, dry solid particles, dispersant, and deionized water were mixed and kerosene was added in proportion followed by sufficient agitation.

### Particle size measurements

Particle size was determined by automatic laser granularity analyzer (Mastersizer 2000, Malvern, UK) by suspending particles in ethanol and subjecting the suspension to ultrasonic diffusion.

### Rheology measurements

Rheological properties of suspensions were measured using a Bohlin CVO rheometer (Malvern, UK) at 25 ± 0.1 °C. The rheometer consisted of a cup centered on a turntable with a rotor concentrically suspended within it. The sample was placed in the annular space between the inner rotor and outer cylinder for measurement. The viscosity of suspensions was measured by logarithmically increasing the shear rate from 0.01 s^−1^ to 100 s^−1^ over a period of 100 s. The value at the shear rate of 100 s^−1^ was used as the apparent viscosity of the suspension. The yield stress is defined as the stress at which the sample begins to deform plastically. Yield stress measurements were performed as follows: a shear stress sweep from high to low stress with 50 sample points over 500 s. Measurements were repeated three times to ensure that the results were reproducible.

### Microstructure of suspension

The microstructures of dried particles were observed using a scanning electron microscope (HITACH SU1510), as shown in Supplementary Fig. S1. The microstructures of suspensions were observed with a CLSM (Nikon A1R). Composite images (Figs 4 and 5, and Supplementary Fig. S7) were created by merging an unfiltered real-light image with a filtered, UV-light image using a hydrophobic fluorescent dye (DiIC1 (5) iodide, Fanbo Biochemical Co., Ltd, Beijing) as a strain in the kerosene mixture. The intensity of the UV-light images was colored red in the composite image for clarity.

### Measurements of contact angle

Solid particles and a certain amount of kerosene were mixed to obtain composite particles, namely particles coated by kerosene. The composite particles were pressed into non-porous discs with a diameter of 13 mm and thickness of 1 mm at a pressure of 12.5 MPa. These discs were stored separately in valve bags to prevent volatilization prior to analysis. Three discs were made for each sample. Three measurements were conducted at different locations on each disc, thus obtaining the mean values of nine measurement results for each particle type.

Contact angles were water contact angles in air, which were measured using a static drop method with an optical tensionmeter (Theta Lite). Photographs were taken at the interface of the disc and water and contact angles were obtained using analytical software.

## Additional Information

**How to cite this article**: Zhang, J. *et al.* Multiple Effects of the Second Fluid on Suspension Viscosity. *Sci. Rep.* **5**, 16058; doi: 10.1038/srep16058 (2015).

## Supplementary Material

Supplementary Information
