# Supplementary material for: Multiple Effects of the Second Fluid on Suspension Viscosity
Source: Sci Rep. 2015 Nov 2;5:16058. doi: 10.1038/srep16058 (PMC4629204; doi:10.1038/srep16058)
Supplement: Supplementary Information [file srep16058-s1.pdf]

**Multiple Effects of the Second Fluid on Suspension Viscosity**

**Authors:** Jie Zhang, Hui Zhao, Weifeng Li, Menghan Xu, Haifeng Liu*

**Supplementary information:**

The images of particles used in this study are shown in Fig. S1.

The structure of MF (dispersant) is shown in Fig. S2.

For PE suspensions, the solid content was 54.2 vol% (53 wt%). The amount of second fluid varied between 0.00 and 2.14 vol%. The effects of the second fluid addition on the rheology of the PE suspension are depicted in Fig. S3.

For BC suspensions, the solid content was 54.5 vol% (63 wt%). The amount of second fluid varied between 0.00 and 3.55 vol%. The effects of the second fluid addition on the rheology of the BC suspension are depicted in Fig. S4.

The real droplet images on PE and BC slices are shown in Fig. S5 and S6, respectively.

Microscopic images of BC suspensions with the second fluid added by different protocols are shown in Fig. S7. Hydrophobic fluorescent dye [DiIC1(5) iodide, Fanbo Biochemicals Co. Ltd.] was used to color the kerosene. The intensity of the UV-light image was colored red in images for clarity.

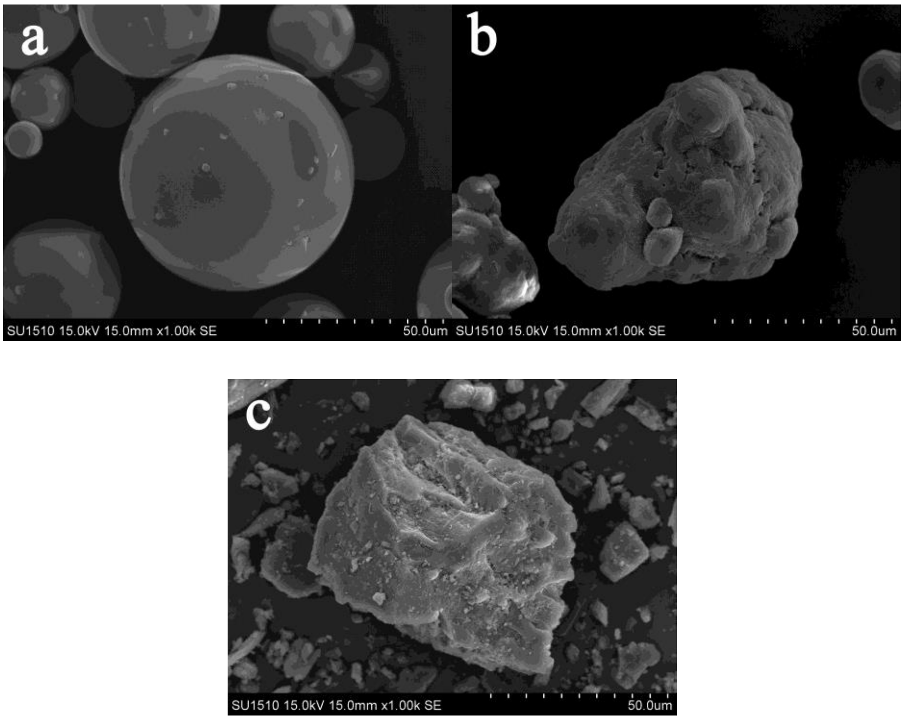


**Supplementary Figure S1 | Microscopic images of the particles used in this study.** (a), SEM image of HGB; (b), SEM image of PE; (c), SEM image of BC.

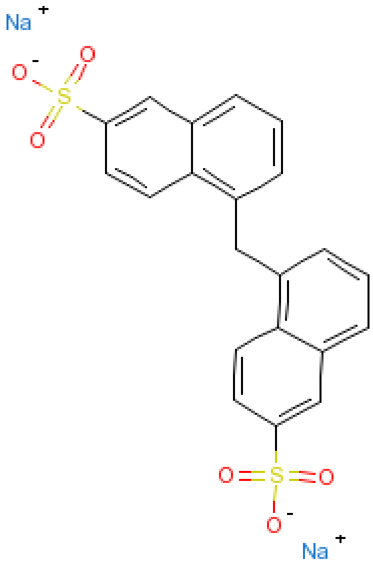


**Supplementary Figure S2 | Molecular structure of MF (dispersant).**

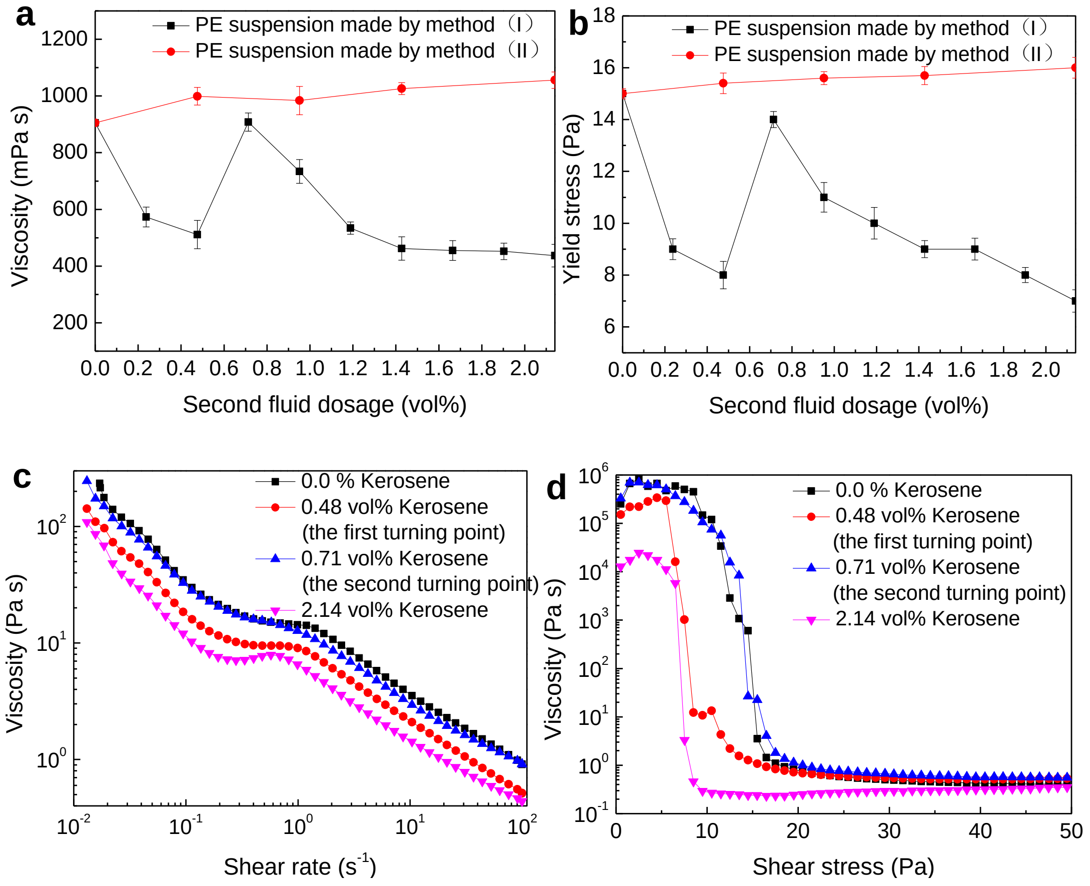


**Supplementary Figure S3| Effect of added secondary fluid on the rheology of PE suspension.** Viscosity (a), at a shear rate of 100 $s^{-1}$, and yield stress (b) trends with various secondary fluid dosages according to different preparation methods. Flow cures [(c) and (d)] by method (I) at the first and second turning points, and a larger dosage of second fluid. Error bars in (a) and (b) indicate repeatability error.

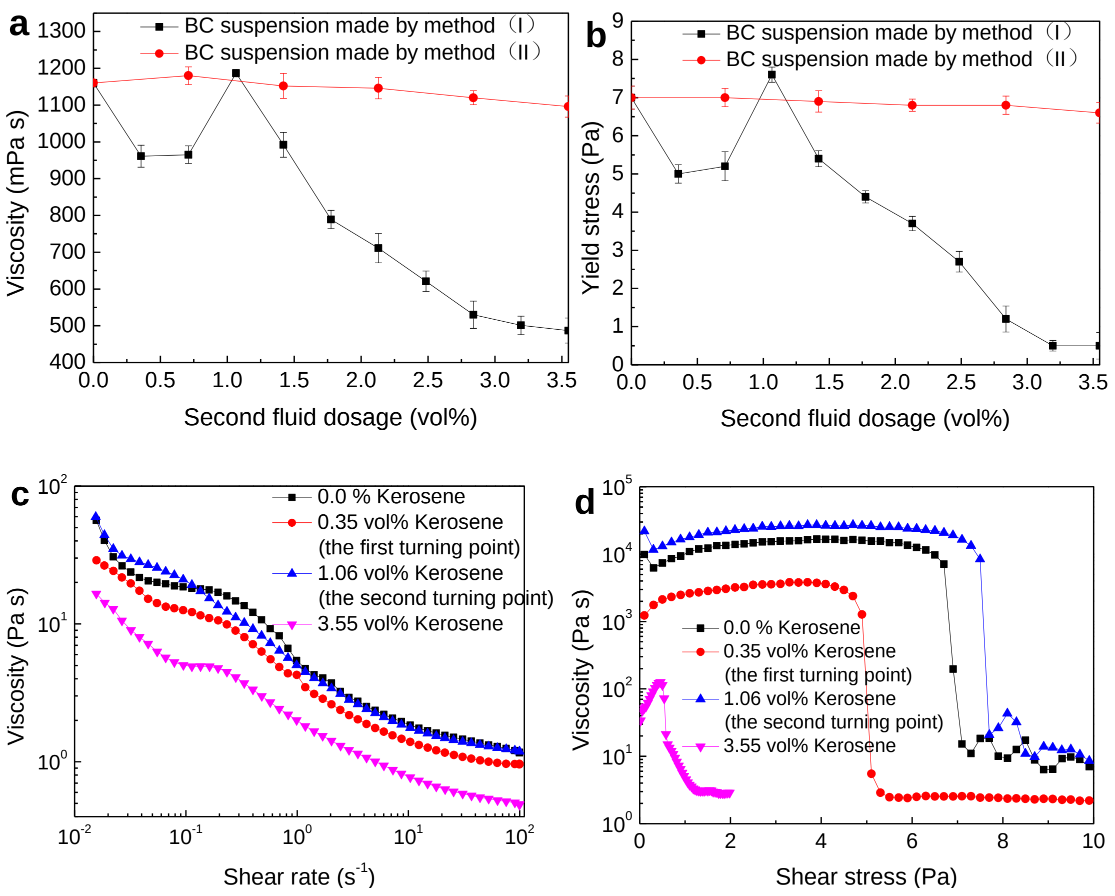


**Supplementary Figure S4| Effect of added secondary fluid on the rheology of BC suspension.** Viscosity (a), at a shear rate of 100 $s^{-1}$, and yield stress (b) trends with different secondary fluid dosages as a function of preparation method. Flow cures [(c) and (d)] by method (I) at the first and second turning points, and a larger dosage of second fluid. Error bars in (a) and (b) indicate repeatability error.

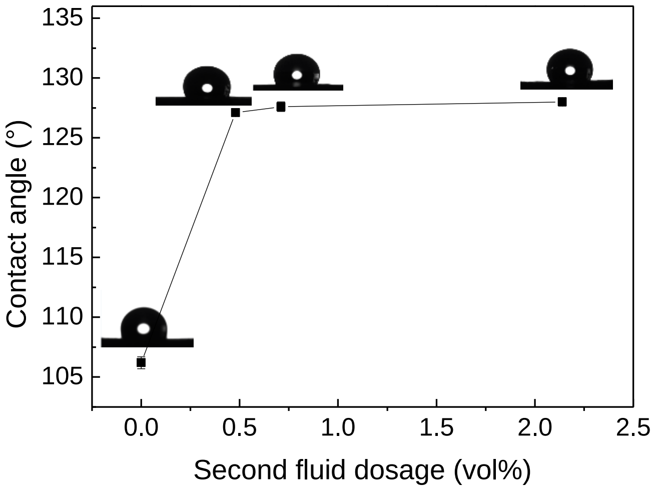


**Supplementary Figure S5| Real droplet images on the PE slices.** PE without modification, θ=106.2 °; at the first turning point, θ=127.1 °; at the second turning point, θ=127.6 °; at one larger dosage of the second fluid, θ=128.0 °. Error bars indicate repeatability error.

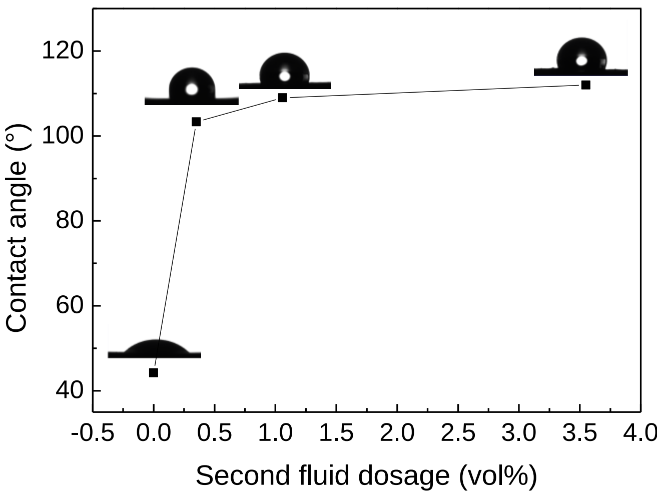


**Supplementary Figure S6| Real droplet images on the BC slices.** BC without modification, θ=44.2 °; at the first turning point, θ=103.3 °; at the second turning point, θ=109.0 °; at one larger dosage of the second fluid, θ=112.0 °. Error bars indicate repeatability error.

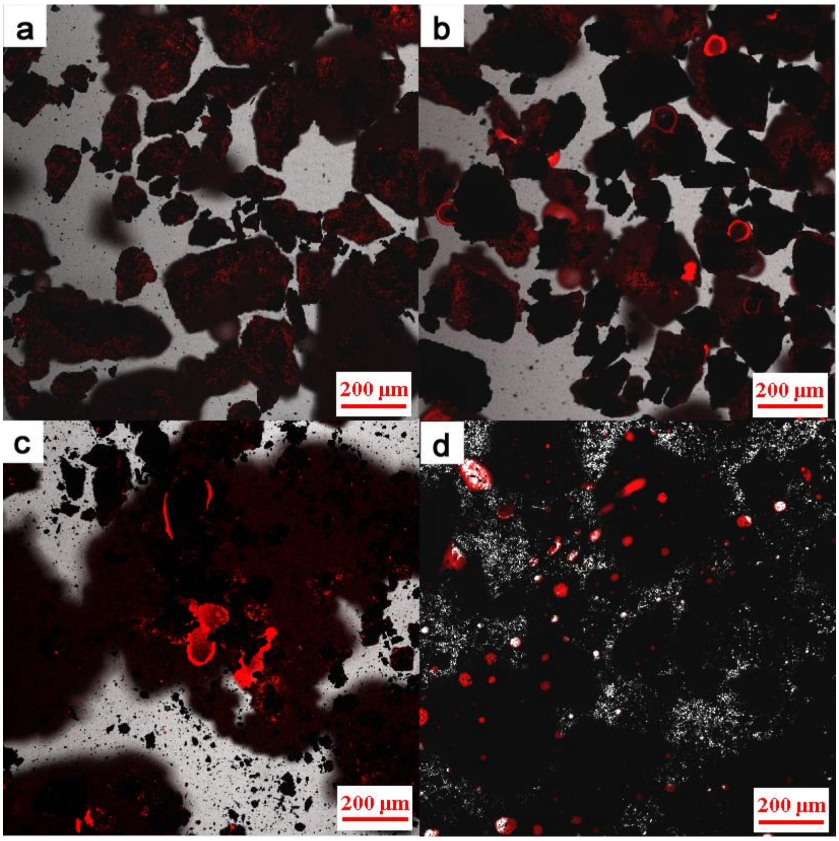


**Supplementary Figure S7 | Microscopic images of BC with the second fluid in different adding methods.** (a), (b) and (c), the first turning point, the second turning point and a larger dosage of second fluid made by method (I), respectively; (d) the suspension made by method (II).
